# A 3-dimensional fibre scaffold as an investigative tool for studying the morphogenesis of isolated plant pells

**DOI:** 10.1186/s12870-015-0581-7

**Published:** 2015-08-26

**Authors:** CJ Luo, Raymond Wightman, Elliot Meyerowitz, Stoyan K. Smoukov

**Affiliations:** Department of Materials Science and Metallurgy, University of Cambridge, 27 Charles Babbage Road, Cambridge, CB3 0FS UK; Sainsbury Laboratory, University of Cambridge, Bateman Street, Cambridge, CB2 1LR UK; Division of Biology and Biological Engineering, and Howard Hughes Medical Institute, California Institute of Technology, Pasadena, CA 91125 USA

**Keywords:** Plant cell culture, 3D culture, Morphogenesis, Scaffold, *Arabidopsis thaliana*, Cytoskeleton, 3D imaging, 4D imaging, Microfibres, Nanofibres

## Abstract

**Background:**

Cell culture methods allow the detailed observations of individual plant cells and their internal processes. Whereas cultured cells are more amenable to microscopy, they have had limited use when studying the complex interactions between cell populations and responses to external signals associated with tissue and whole plant development. Such interactions result in the diverse range of cell shapes observed *in planta* compared to the simple polygonal or ovoid shapes *in vitro*. Microfluidic devices can isolate the dynamics of single plant cells but have restricted use for providing a tissue-like and fibrous extracellular environment for cells to interact. A gap exists, therefore, in the understanding of spatiotemporal interactions of single plant cells interacting with their three-dimensional (3D) environment. A model system is needed to bridge this gap. For this purpose we have borrowed a tool, a 3D nano- and microfibre tissue scaffold, recently used in biomedical engineering of animal and human tissue physiology and pathophysiology *in vitro*.

**Results:**

We have developed a method of 3D cell culture for plants, which mimics the plant tissue environment, using biocompatible scaffolds similar to those used in mammalian tissue engineering. The scaffolds provide both developmental cues and structural stability to isolated callus-derived cells grown in liquid culture. The protocol is rapid, compared to the growth and preparation of whole plants for microscopy, and provides detailed subcellular information on cells interacting with their local environment. We observe cell shapes never observed for individual cultured cells. Rather than exhibiting only spheroid or ellipsoidal shapes, the cells adapt their shape to fit the local space and are capable of growing past each other, taking on growth and morphological characteristics with greater complexity than observed even in whole plants. Confocal imaging of transgenic *Arabidopsis thaliana* lines containing fluorescent microtubule and actin reporters enables further study of the effects of interactions and complex morphologies upon cytoskeletal organisation both in 3D and in time (4D).

**Conclusions:**

The 3D culture within the fibre scaffolds permits cells to grow freely within a matrix containing both large and small spaces, a technique that is expected to add to current lithographic technologies, where growth is carefully controlled and constricted. The cells, once seeded in the scaffolds, can adopt a variety of morphologies, demonstrating that they do not need to be part of a tightly packed tissue to form complex shapes. This points to a role of the immediate nano- and micro-topography in plant cell morphogenesis. This work defines a new suite of techniques for exploring cell-environment interactions.

**Electronic supplementary material:**

The online version of this article (doi:10.1186/s12870-015-0581-7) contains supplementary material, which is available to authorized users.

## Background

Studies of plant development aim to understand processes that occur from the molecular scale through to the cellular and tissue scales, to the organism as a whole. Such studies routinely make use of live imaging, combined with transgenic modifications to introduce fluorescent reporters for observing a process of interest. For studying multicellular interactions and morphogenetic processes, imaging makes use of whole plants or tissue explants, yielding useful information for both the complete structure and the influence this structure has on the molecular processes within the cells. Single, isolated cells permit easier access to the subcellular dynamics, especially for cell types that are poorly accessible or difficult to orient for imaging. It is, however, difficult to isolate processes on the single cell-scale whilst concurrently maintaining the tissue-scale response to external signals from a 3D environment. This makes a new model system based on cultured cells interacting within a tissue-like scaffold a desirable biological tool.

Current plant cell methodologies place cultured cells mostly on flat, two-dimensional (2D) surfaces (microscope slide, bottom of a culture dish) where they cannot interact with 3D environments. One exception is the use of lithographically defined microfluidic channels that have been useful tools for determining the behaviour of pollen tube growth in response to controlled chemical gradients and mechanical obstacles [1, 2]. Microfluidic methods have high potential to provide single cells with defined quantities of diffusive signals and a confined environment akin to that of plant cells *in vivo*, however, microfluidic devices at present do not integrate 3D tissue-structures (scaffolds) in the confined environment to better mimic native tissue conditions.

Human tissue engineering employs 3D scaffolds mimicking the extracellular matrix (ECM) to provide a tissue-environment and this culture method of animal cells *in vitro* are the subject of intense development [3, 4]. The design and engineering of suitable scaffolds that capture the complex *in vitro* 3D physiology have been refined over the last 20 years [5]. An optimised scaffold should provide micropores that permit cell penetration, a biocompatible nano-topography and fibres with tuneable tissue-specific mechanical properties. Polymeric microfibres can give a scaffold cell-size pores and a broad range of mechanical strength but cannot provide the nano-topography required for cell attachment; whereas polymeric nanofibres alone can provide ECM-mimicking and biocompatible nano-topography but are limited in the achievable range of mechanical properties and pore sizes required for different cell types. Hence, alternating layers of nanofibres and microfibres is a major strategy for constructing tissue scaffolds [6–8]. Commercial 3D printing still does not have the resolution for fine tissue patterning, and combining it with nanofibres in a single process has been a challenge [7]. The combined processes cannot achieve a scaffold that is profitable to manufacture at an industrial scale whilst providing the desirable micro- and macroscopic properties.

Shear spinning is a recently commercialised technology (www.xanofi.com) that can achieve high-yield production of integrated micro- and nano-fibre scaffolds with an appreciable thickness (up to several centimetres) necessary for the 3D cell models [9, 10]. The process extrudes and shears a polymer solution in a non-solvent and is able to produce continuous or staple nanofibres or microfibres, that can be mixed and dried to form scaffolds of various density and porosity [9, 11]. While such scaffolds are emerging in the study of mammalian biology, their suitability for fundamental plant biology has not been explored.

This study applies 3D tissue engineering to the plant sciences and reports (1) the development of an effective protocol for plant cell culture in scaffolds; (2) the characteristics of the scaffold required for optimal plant cell attachment; (3) the influence of the scaffold structure on cell morphology; (4) the potential to study physiological responses to phytohormones. We make use of commercially available and cost-effective shear-spun 3D scaffolds, constructed from a mix of biocompatible poly(ethylene terephthalate) (PET) microfibres and polylactide (PLA) nanofibres. These allow imaging of cells with high spatial resolution similar to that in other single cell studies, but in a 3D fibrous environment mimicking the extracellular matrix. The cells display morphologies previously not seen in cultured cells and not normally visible *in planta*, while at the same time enabling us to record 3D and 4D data of cell growth and cell-environment interactions. We demonstrate these advantages using a fast protocol of seeding callus-derived liquid cultures of the laboratory model plant *Arabidopsis thaliana* in the scaffold. We show evidence of specific adhesion interactions of the cells to the scaffold, which likely influence the growth and geometry of the cells. This work defines a new suite of techniques for the growth and time-lapse imaging of plant cells interacting with each other and with tissue-like environments.

## Results

### Seeding fibres using liquid culture cells derived from seed calli

**Fig. 1:**
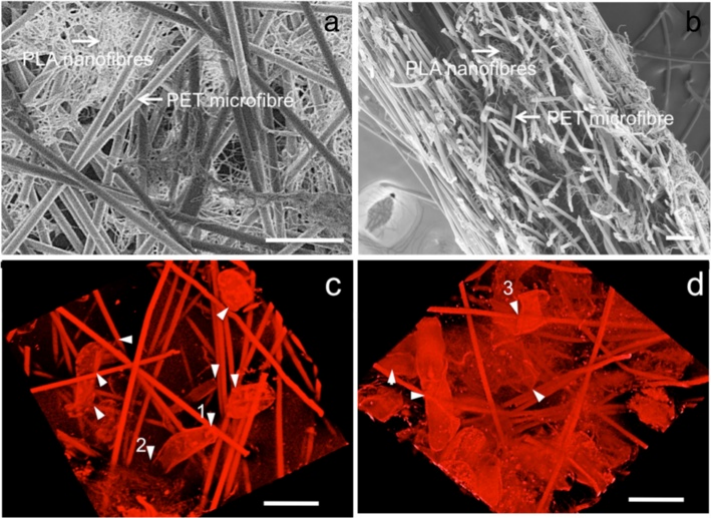
Scanning electron microscopy (SEM, a-b, greyscale) and confocal images (c-d, false colour red) showing the 3D polymer scaffolds and *Arabidopsis thaliana* cell growth in the scaffolds. **a**-**b** SEM images of 30 % PLA nanofibres, 70 % PET microfibre scaffold before cell seeding: **a** front-view, **b** side-view. **c**-**d** 3-dimensional reconstructions of confocal z-stacks showing cells of *Arabidopsis thaliana* expressing a reporter construct expressing cytoplasmic mCherry: **c** day 1 and **d** day 4 growth of cells inside the scaffold. Proliferation and growth were observed throughout the scaffold. Cells increased in number and size from day 1 to day 4. Cells formed local points of attachment on the fibres (*arrows*) and subsequently expanded in size into the porous space either by stretching from or winding around microfibres. For example, *arrows 1* and *2* point to a cell attached to a microfibre at point *1* and growing into the depth of the fibrous scaffold as shown at point *2*. *Arrow 3* highlights a cell wrapping around a microfibre. Scale bars: 100 μm

Arabidopsis transgenic seeds are induced to form calli. *Arabidopsis* transgenic lines, containing various fluorescently labelled reporters, can be readily prepared as a cell suspension in as little as 7–14 days (see Methods), by using a defined medium containing phytohormones. The suspension cultures contain a large proportion of single cells compared to clumps. Cultures are used to seed pre-wetted scaffolds consisting of PET (microfibres) : PLA (nanofibres) in a ratio of 70 % : 30 %. The scaffolds are organised as a layered-meshwork of the PET microfibres incorporating the finer PLA nanofibres (Fig. 1a-b). Cells expressing cytoplasmic mCherry are seeded on the scaffolds and visualised with a confocal microscope, where the PET microfibres are also visible due to their auto-fluorescent signal at wavelengths above 600 nm (Fig. 1c-d). Scaffolds are capable of maintaining cell growth and morphogenesis for 72 hours after seeding without further manipulation. By replacing the culture media daily after 72 hours of seeding, cells may be maintained within the scaffold beyond 10 days (Additional file 1: Figure S1).

## Developing an effective sterilisation procedure for routine use of the 3D scaffolds

The protocol to sterilise the scaffolds before coming into contact with the sucrose-containing suspension medium is important to prevent fungal contamination. Sterilisation techniques by ethanol, ultraviolet (UV) irradiation and X-ray irradiation have been tested. Additional file 1: Figure S2 shows the morphology of the scaffold before and after various sterilisation treatments. X-ray sterilisation is the most effective method. X-Ray sterilisation for up to 18 minutes at 417 cGy/min irradiation results in no appreciable change of fibre morphology (Additional file 1: Figure S2). UV irradiation has been the most common practice for sterilising nanofibre-scaffolds. However, for thicker 3D constructs used in this work, at 0.78 ± 0.07 mm average scaffold thickness, UV light fails to penetrate the centre of the scaffold and frequent fungal contamination originates from this region. Ethanol-treated scaffolds do not allow cell growth and PLA nanofibres appear fused. Ethanol is a nonsolvent of PET but a poor solvent of PLA. Hence, the reasons of poor cell growth on ethanol-sterilised scaffolds may be two-fold: (1) ethanol renders the scaffold morphology unsuitable for cell attachment; (2) the *Arabidopsis* cell cultures are sensitive to residual ethanol. In addition, we note that ethanol sterilisation is also ineffective against bacterial contaminations [12].

### Plant cells interact with the scaffold components

Cells appear to have fixed positions in the scaffold and do not exhibit Brownian movements within the field of view (x, y or z dimensions) during microscopy whether they are larger or smaller than the pores created by the fibres around them. Cells remain fixed in the structure after the cell-seeded scaffolds are transferred to fresh media and agitated at 130 rpm for 60 minutes (Additional file 1: Figure S3). Furthermore, cells are observed to be in contact with the microfibres (Fig. 1c, white arrows).

To determine whether the cell-fibre attachments are active cellular interactions with the artificial structure or simple passive entrapment of cells by the porous scaffold, we have repeated the cell culture experiment in the scaffold using fluorescent silica particles of similar size and concentration to the *Arabidopsis* cells in suspension. The particles have a size range of 40–200 μm that resemble the size range of *Arabidopsis* cells. We observe that the silica particles become passively trapped in the scaffold, which acts as filters, but the particles readily detach from the scaffold. By analysing scanning electron microscope (SEM) images (Additional file 1: Figure S4) and counting the number of silica beads on the scaffold surface, we find approximately 94 % of the silica beads filtered in the scaffold have detached from the scaffold after agitation in the cell culture medium at 130 rpm.

The adherence of the cells to the fibres is not due to excess mucilage released during cell culture from the seed-derived calli. Stable *Arabidopsis* cell culture lines not derived from seed also grow in the scaffold and interact with the fibres. Both seed-derived and non-seed derived cells exhibit similar behaviour of winding and twisting around microfibres as observed by light microscopy (Additional file 1: Figure S5), demonstrating that cell-scaffold interactions are not due to seed mucilage.

**Fig. 2:**
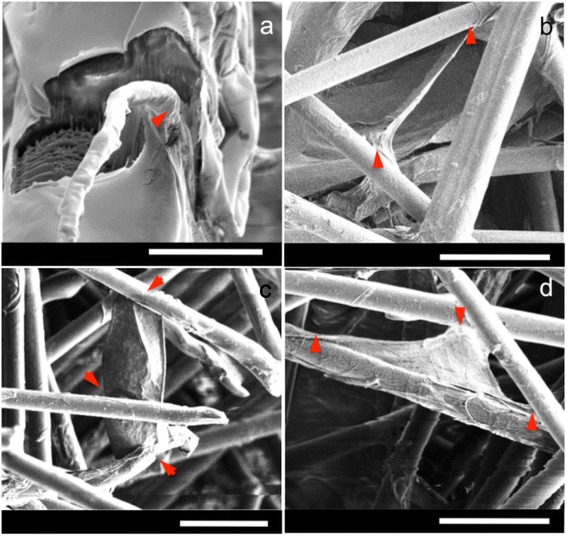
**a** SEM image of a cross-section of a cell on top of a microfibre sliced by a focused ion beam, showing the attachment of the cell to a nanofibre (*red arrow*). The surface of the cell, attached to the fibre, is shown by a *red arrow*. Internal cellular structures have been exposed after ion beam milling. **b**-**d** SEM images under high vacuum conditions showing strong cell-fibre attachment to surrounding fibres, indicated by *red arrows*. Scale bars: **a** 10 μm, **b**-**d** 50 μm

**Fig. 3:**
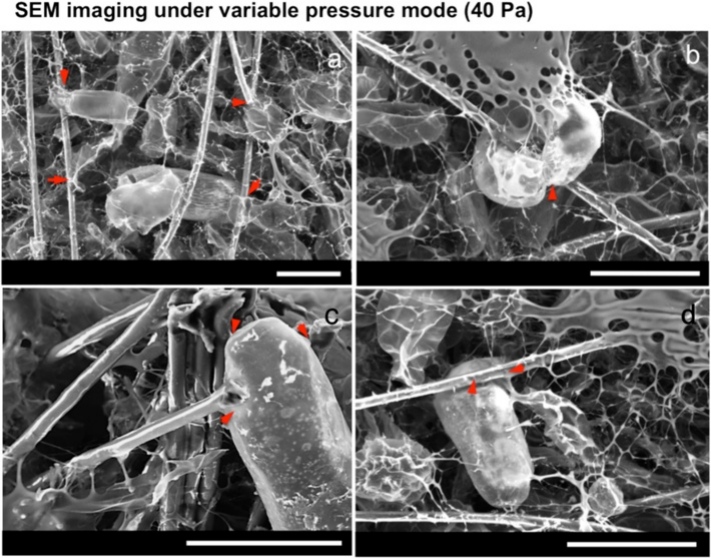
Variable pressure SEM images obtained at 40 Pa, showing cell-fibre interactions in the scaffold. Local points of attachment between the cell wall and the fibres are highlighted by *red arrows*. **a** An overview of cells in scaffold. **b**-**d** Images of single cell-fibre interactions.Scale bars: 100 μm

**Fig. 4:**
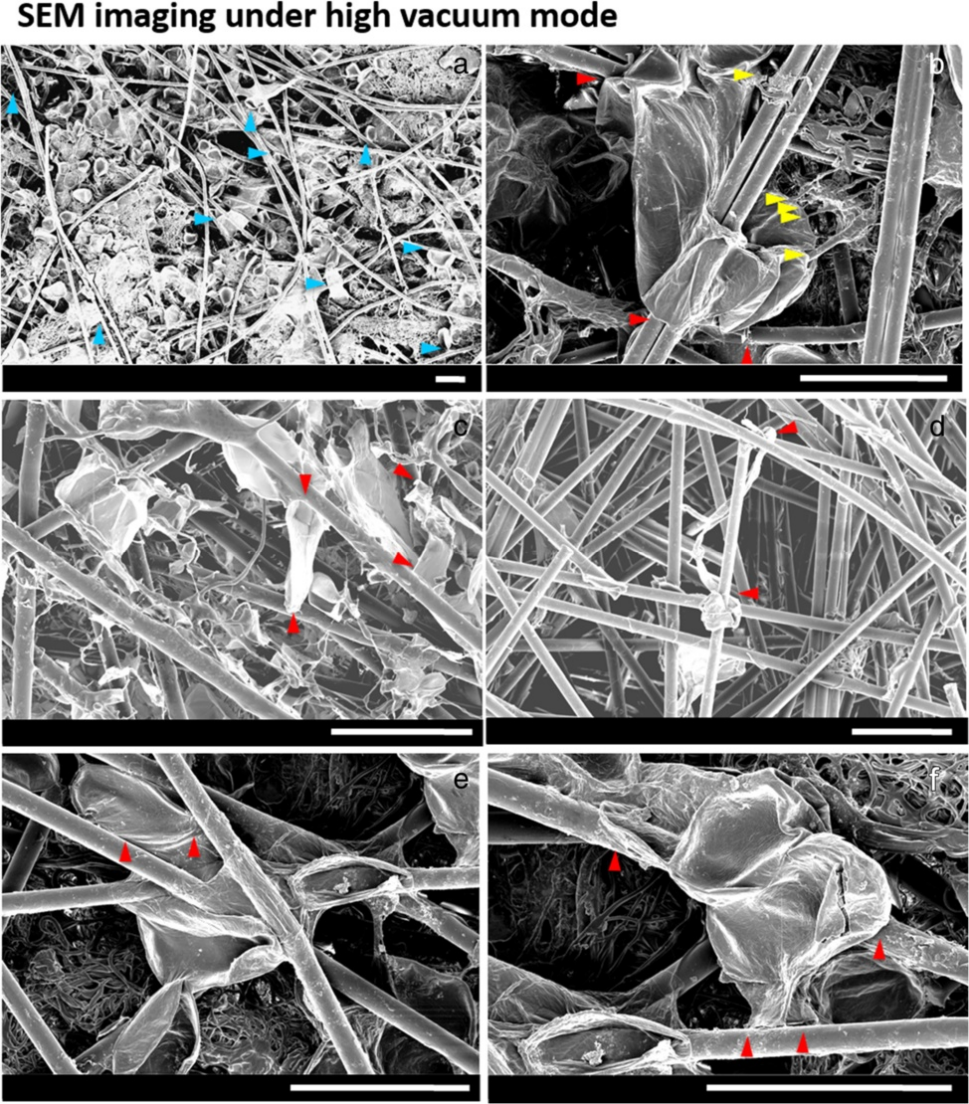
SEM images obtained under high vacuum conditions showing cell-fibre interactions in a 3D microenvironment. Attachment points between the cell wall and the micro- and nanofibres are highlighted by *red* and *yellow arrows*, respectively. **a** Overview of the abundant presence of cells in the scaffold. Examples of cells are indicated by *blue arrows*. **b**-**f** Images of cells winding around or reaching between microfibres (*red arrows*) with direct attachment to nanofibres (*yellow arrows*). As the cell deflated under vacuum, the cell wall pulled back with parts of the cell remaining attached to the fibres. Scale bars: 100 μm

Microfibres can be clearly imaged using confocal microscopy but nanofibres cannot be visualised. To understand cell-nanofibre interactions, a focused ion beam is used to remove part of the cell surface during SEM, showing a cell adapting its shape to enclose a nanofibre (Fig. 2a-b). SEM experiments are done under both variable pressure (Fig. 3) and high vacuum modes (Fig. 2 and Fig. 4). Under variable pressure SEM mode, moist samples are imaged at 40 Pa and cells deflated gradually over several minutes. Cell-fibre attachments are observed and remain constant (Fig. 3). When the SEM mode is changed from variable pressure to high vacuum mode, cells deflate but remain attached to the scaffold. Yellow arrows in Fig. 4b reveal the firm focal attachment between the deflated cell and the neighbouring PLA nanofibres. The cell remains wound around microfibres (red arrows). In examples shown in Fig. 4c and d, where cells do not wind around a fibre, but reach between two fibres, the deflated single cell with no other support does not detach from cell-fibre focal points (red arrows) and remains immobilised like a bridge between two microfibres. Another example of a cell bridging gaps between microfibres is shown in Fig. 1c.

**Fig. 5:**
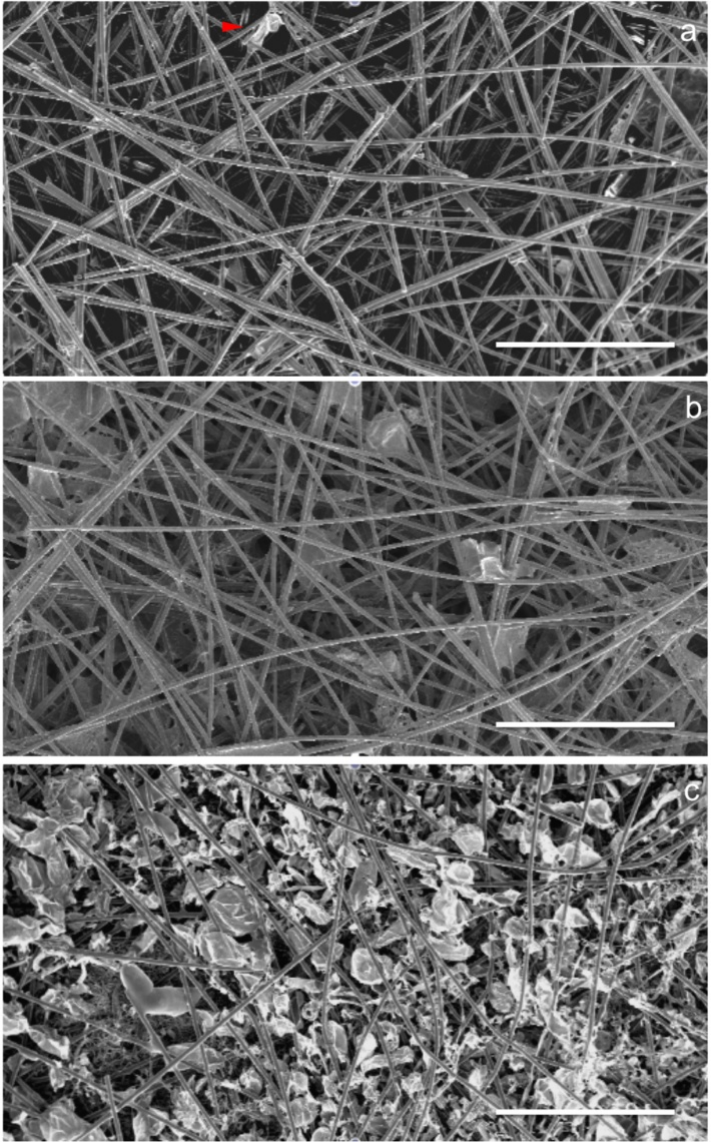
**a**-**c** SEM images at day 3 after seeding cells in scaffolds of varying nanofibre percentage. **a** No PLA nanofibres, 100 % PET microfibres. Few cells grew on the scaffold, though a cell can be observed to interact with a PET microfibre (*red arrow*). **b** 10 % PLA nanofibres, 90 % PET microfibres. **c** 30 % PLA nanofibres, 70 % PET microfibres. Compare the Scale bars: 500 μm

The observations that (1) cells of diverse shape and size are immobilised, and (2) cells maintain contact with one or more fibres upon application of force, suggest a physical interaction between the cells and the fibres is more consistent with active adherence rather than passive entrapment. As evidenced by the adherence interactions above, the fibrous scaffold is able to provide a three-dimensional support for plant cell culture growth and morphogenesis. Plant cells respond to nanofibre concentration in the scaffold in a similar fashion to that observed in mammalian cell culture [7, 13], in which the initial cell attachment density increases with increasing nanofibre percentage in the scaffold. Specifically, cell count increases from 5.4 ± 4.4 cells/mm^2^ for 0 % nanofibres, to 12.6 ± 3.6 cells/mm^2^ for 10 % nanofibres, to 93.5 ± 58.9 cells/mm^2^ for 30 % nanofibres (Fig. 5). All scaffolds contain the same mass of PET with increasing mass of PLA nanofibres. Compared to the PET microfibres, PLA nanofibres can be described as a more voluminous and tufted material. This led to an increase in the thickness of the scaffold per unit area with an increasing PLA content, but also resulting in an overall relatively unchanged porosity value (68 ± 1 %, see Methods) for all scaffolds despite the changing nanofibre content. Hence, the increasing cell seeding density with respect to nanofibre percentage in the scaffold is not due to changes in porosity of the material that may change the space available for cell attachment and growth. In addition, *Arabidopsis* cells appear to adhere with nanofibres at the cell surface, and continue to conform and adapt their shape and orientation according to the adjacent microfibres.

### Cells interact with scaffolds and display shapes not usually seen *in planta*

**Fig. 6:**
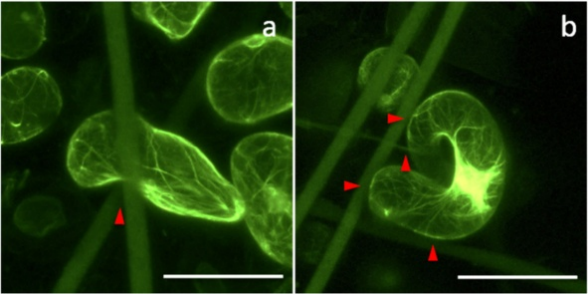
Confocal images of actin-labelled *Arabidopsis* cells expressing the reporter construct *35S::GFP-FABD2*, showing the actin patterns in growing cells and the orientation of cells as they interact with the scaffold. **a** A pinched cell expanding. Microfibres exist in front of and behind the constriction point (*arrow*) **b** Spiral shape of cell as it attached, interacted and wound around fibres inside the scaffold. *Red arrows* indicate points of cell-fibre interactions. The large mass of actin corresponds to the nuclear basket. Scale bars: 100 μm

**Fig. 7:**
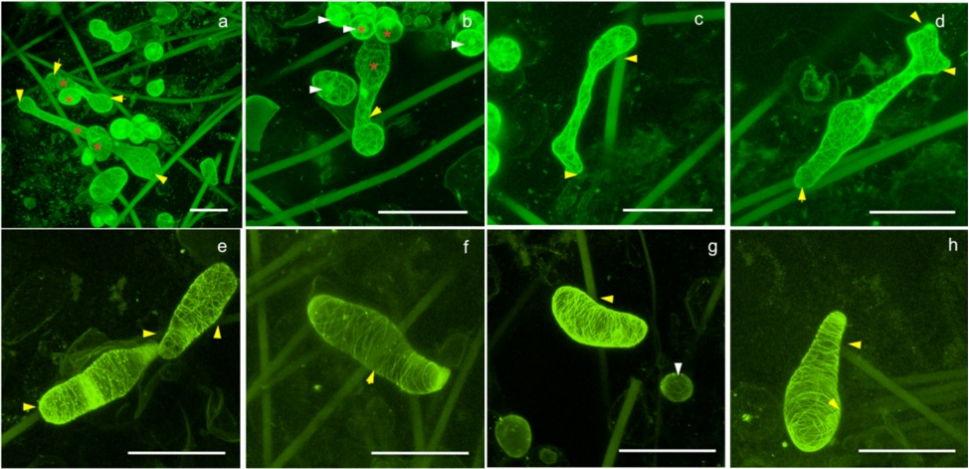
Confocal z-projections showing cells adapting their shape to interact with the fibrous environment. **a** An overview. **b**-**h** Higher resolution examples of cell shapes.*White arrows* indicate small round cells. *Yellow arrows* indicate cell-fibre interaction. Red asterisks in **a**-**b** indicate heterogeneous growth between neighbouring cells, demonstrating the ability of the cells to slip past each other and continue elongation, a behaviour unobserved in native tissues. GFP-labelled microtubules in cells expressing reporter construct *35S::GFP-MBD*. Scale bars: 100 μm

**Fig. 8:**
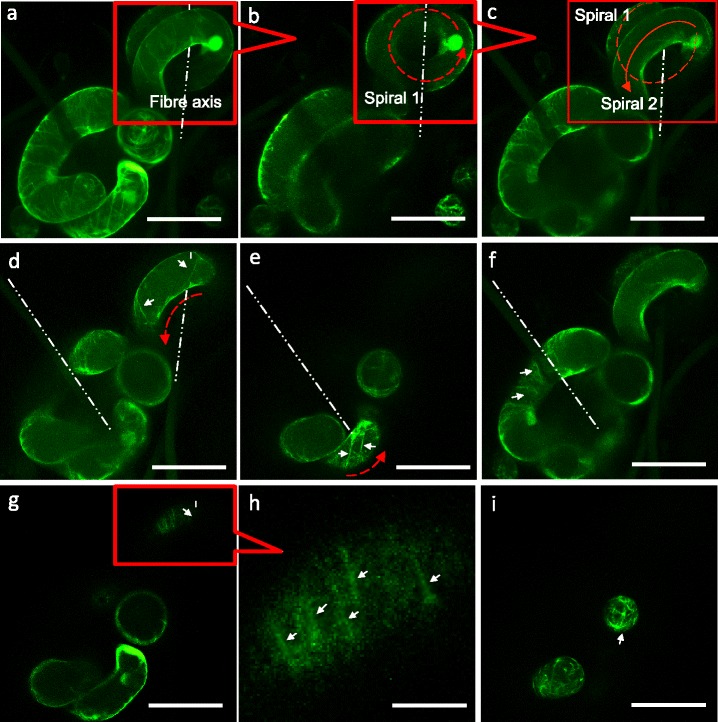
Confocal z-projections showing GFP-labelled microtubule arrays in *A. thaliana* cells expressing reporter construct *35S::GFP-MBD*. *White arrows* indicate microtubules. Dotted lines trace fibres. **a**-**c** A cell spiraling twice around a microfibre. **d**-**f** Diagonal microtubules in spiral cells bending around the central axis. **g**-**h** Conventional microtubule patterns perpendicular to direction of elongation. **i** Radial/criss-cross pattern of microtubules in small round cells (diameter < 50 μm) and the tips of elongating cells. Scale bars: 100 μm

Large cells are found to grow adjacent to, between and around microfibre supports, as well as across several microfibres. Cultured and newly seeded cells commonly exhibit shapes that are round, elongated-straight or elongated-arced. Adhered cells can be seen to exhibit anisotropic expansion, growing between gaps within the fibre. Where gaps are narrow, cells appear to alter their shape to continue growth and the regions in narrow gaps appear as constricted regions along the length of the cell. For example, where parts of the cells seem severely restricted and “pinched” between two microfibres (Fig. 6a), the rest of the cell appears to have expanded and explored new space. Cells can also be found to occupy space along the length of the same microfibre (Fig. 7e). 48–72 hours after seeding, cells are seen to be very elongated, with numerous examples of spiral-shaped cells around microfibres (Fig. 6b, Fig. 8 and Additional file 1: Figure S6). As cells grew much larger they are seen to adopt more complex shapes (Fig. 7 a-d). Cells remain immobilised inside the scaffold when we vary the vacuum condition from variable pressure to high vacuum using a variable pressure SEM. These extreme geometries and orientations of very long and twisted cells are not present in the culture at the time of seeding.

### Cytoskeletal organisation in response to cell-fibre interactions

Control of plant cell expansion requires the correct deposition of cell wall material, which is influenced by the arrangement of the underlying cortical cytoskeleton formed of microtubules and actin. In longitudinally (anisotropically) expanding cells, for example in hypocotyl or root epidermal cells, actin appears as a complex network of thick bundles or narrow fibres found in various orientations within a single cell and the actin network has been shown to transport the Golgi apparatus and various types of post-Golgi compartments that contain cell wall material [14, 15]. Live observations of actin can be carried out using confocal microscopy of a GFP fusion with a portion of the *Arabidopsis* Fimbrin1 protein (called GFP-FABD2). At sites of apparent space constriction, or where the cell interacts with a fibre, actin can sometimes be observed to bundle as shown in Fig. 6a, where intensely fluorescent actin is observed close to the intersection of two microfibres (red arrow). Figure 6b shows actin in a cell undergoing spiral growth, where long actin filaments emanating from the ends of the cells appear to converge on the nuclear basket. These observations may reflect local differences in transport of wall material to achieve a shape change.

We next looked at microtubules in cells expressing a fusion between GFP and the microtubule-binding domain of the mouse MAP4 protein. In cells exhibiting anisotropic expansion, microtubules are observed to orient perpendicular to the long axis (Fig. 7f-h) – consistent with their role in directing cell reinforcement by influencing cellulose deposition [16]. In ovoid (non elongating) cells and in cells exhibiting complex shapes (large cells in Fig. 7a-d), microtubules orientations are not transverse to the long axis (red asterisk in Fig. 7a-b and Additional file 2: Movie S1). An enlarged view of a highly elongated portion of an irregular shaped cell is shown in Additional file 1: Figure S7, in which microtubules are oriented predominantly along the long axis. In the ovoid portions of the same cell, the microtubules exhibit a mesh-like configuration. In cells growing in spirals around individual fibres (Fig. 8), microtubules are often arranged diagonally, except for the ends of the cells that, when viewed faced on, adopt the mesh-like configuration. Unlike natural tissues, in which cells cannot grow past each other and often show homogeneous growth between neighbouring cells, the single cells in the scaffold show heterogeneous growth between adjacent cells. Larger, elongating cells are capable of growing past fibres and other obstructing cells to fill the available space (e.g. long cell in Fig. 7a and b). As a proof-of-concept we could track the growth and catastrophes of individual microtubules in a 4D data series (Additional file 3: Movie S2). Further work based on the 3D cell culture method reported in this work will correlate microtubule orientations and cell wall formation in *Arabidopsis* cells interacting with the 3D environment over time.

### Applicability to other cell lines

**Fig. 9:**
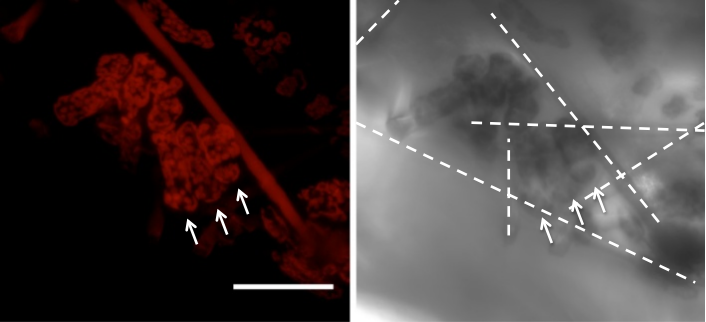
Average projection of images of *Z. elegans* cells taken 3 days after seeding in scaffolds. Shown are autofluorescence in the red spectrum (*left panel*) and the corresponding transmission micrograph (*right panel*). Locations of fibres are marked as *dashed lines*. Alignment of cells in a confined space is indicated by *arrows*. Scale bar: 100 μm

The 3D scaffolds are applicable to studying cells of species besides *Arabidopsis*. We cultured mesophyll cells of *Zinnia elegans* inside the scaffold. When cultured in “non-inductive medium”, where cells do not differentiate into tracheary elements, *Zinnia* cells continually exhibit growth [17]. By imaging autofluorescence of the wet cell-seeded scaffold, *Zinnia elegans* cells are observed to grow along the fibres, and fewer cells are found in spaces without the fibres (Additional file 1: Figure S8a-b). High vacuum SEM (c, d) reveals regions of high density cell seeding, together with apparent attachment points as previously found for the *Arabidopsis* cells. The high density regions permit a closer look at cell-cell interactions that are more akin to native tissue conditions, in which cells are tightly packed. In a confined space delimited by fibres (Fig. 9), three cells of similar size line up next to each other and maintain contact along their long edges. This contrasts to what we have seen in *Arabidopsis* where neighbouring cells grow past each other (Compare with Fig. 7a).

### Encapsulating plant growth substances within the scaffold fibres

In mammalian 3D cell culture, hormones can be encapsulated in polymeric scaffolds for sequential and timed release of implanted bioactive agents [18]. Auxin is a principal regulator of growth and pattern formation in plants. The synthetic auxin, 2,4-dichlorophenoxyacetic acid (2,4-D), is readily soluble in organic solvents that facilitates its incorporation during the formation of the scaffolds. Briefly during scaffold fabrication, 0.5 % w/w of 2,4-D is dissolved in a 15 % w/w PLA solution and the mixture is shear-spun to form a fibrous PLA scaffold (see Methods and ref [10]). Although the release profile of auxin from the scaffold fibres is unknown, we find 5 % w/w 2,4-D or higher incorporation in the fibre-forming solution results in rapid cell death, consistent with its herbicidal properties. At 0.5 % w/w 2,4-D in the polymer solution, cells on the resulting fibres can be maintained for up to 3 days. As relatively small amounts of auxin are already present to maintain plant cells in culture, it is not immediately apparent if there is any physiological response to the scaffold-released auxin. The *DR5::GFP* construct has been used in BY2 cells, encoding a marker to visualise auxin uptake activity [19]. In our work, *Arabidopsis DR5::GFP-ER* yields a signal in some cells within the liquid cultures, consistent with *DR5* response to the exogenous 2,4-D. We observe no morphological responses of the cells to the extra auxin released from the scaffold during the 3-day period of cell culture, however, after 48 hours no GFP signal is observed for cells seeded in the scaffold without the encapsulated auxin, whereas the DR5 GFP signal is maintained within the auxin scaffolds (Additional file 1: Figure S9). Auxin efflux carriers, encoded by *PIN* genes, are known to be polarly localised [20]. A polar distribution of PIN indicates the presence and propagation of auxin gradients. PIN7-GFP is observed in some liquid cultured cells as discrete punctae representing intracellular vesicles, however, this pattern of localisation remains unchanged in the auxin scaffolds (Additional file 1: Figure S9), suggesting that either no microgradients exist or the isolated cells are unable to detect or respond to these gradients. We found no detectable signal from a fusion between the principal efflux protein PIN1 and GFP either in liquid culture or the cultures where 2,4-D is released from the scaffold, suggesting PIN1 is not expressed in these types of cultured cells.

## Discussion

A number of technologies are shared in plant and animal biotechnology. Recent efforts with plant cells have used lithographically defined structures to clarify the behaviour of pollen-tube growth and microfluidic systems in general have allowed the control of local chemical gradients and study of interactions with mechanical obstacles [1, 21–23]. Both lithography and microfluidic systems are well known systems for mammalian cell biology in healthcare applications such as diagnostics, cancer research and regenerative medicine [3, 24–26]. Microfluidic systems provide cells with a confined and well controlled environment akin to the *in vivo* plant tissue environment, but lack the structural sophistication of a tissue environment. The 3D cell culture method presented here is a complementary method useful for studying morphological changes of isolated cells that interact with an extracellular structure. Future work incorporating 3D scaffolds in a microfluidic device may enable a better biomimetic environment.

Other methods and materials, such as 3D scaffolds in tissue engineering and regenerative medicine of animal cells have potential applications in plant sciences. We have demonstrated that 3D nano- microfibre scaffolds can be applied as an effective tool for studying plant cell morphogenesis and can help identify new capabilities of growth at the cellular level. The scaffold materials, PLA and PET, are both hydrophobic and require pre-wetting of the fibre scaffold in culture media prior to use. Despite the hydrophobicity, cells are completely immobilised within the scaffold, and focal points (suggesting cell-fibre attachment) are observed with the SEM. Further studies will investigate if the attachment is through adhesion, by investigating known components such as the type and distribution of pectin. If attachment is found to be through adhesion, it would suggest cells of land plants have retained the ability to adhere to relatively inert supports, much like single-celled organisms from their ancestral lineage, represented by the green algae of the charophyta [27, 28]. A recent report has found similarities in composition and structure between the adhesive matrix of Penium, a charophytic green alga, and the middle lamella of land plants that permits the integration of cells into complex tissues [27]. One biological focus of future work is to determine whether the interface between cell-fibre contacts is similar to those of cell-cell contacts.

Shapes of cells in scaffolds range from regular ovoid or cuboid to complex shapes with constrictions where local space appears limited, some even have projections resembling, to some degree, the lobes of leaf pavement cells (Fig. 7d upper right portion of the cell). Coupled with these complex shapes, growth resulted in large sizes of some cells (500 μm in axial length). This may be due, in part, to having a tissue-like environment without the constraints of tightly packed or attached cells and we envisage that the scaffolds will help us determine the factors that govern the upper size limit of a plant cell, a subject of recent discussion [29].

Long cells often grow and orient along the microfibres in either a left-handed or a right-handed spiral conformation. We assume that spiral growth is akin to growing along a flat surface but, given that the diameter of the cell (>40 μm) is many times that of a microfibre (10 μm), the cells grow around the support. To maintain such spiral growth, the cell would likely adhere to the microfibre. It seems likely that the stiffness of the microfibre also contributes to cell growth, and this may be why we do not see spiral morphologies associated with nanofibres. The interaction and adhesion between cells and the microfibres are most likely due to cell wall-fibre interactions. The cell wall defines plant cell shape, which is dependent on the balance between turgor pressure and the structure and composition of the cell wall that is in a constant flow of synthesis and remodelling, based on the type and developmental stage of the cell [30]. For a cell to change its shape, the cell wall must first undergo controlled and sometimes local loosening [31]. A number of experimental studies indicate that a sensing and signalling system exists in the plant that monitors the structure and integrity of cell walls [30]. The new method of 3D plant cell culture reported here has potential to explore the relationships between signalling, synthesis and remodelling of the wall through genetic strategies such as use of existing collections of *Arabidopsis* insertion (mutant) lines and more reporter constructs. Future work will make use of other materials that are bio-inert or bioactive, including cellulosic fibres, to further study the cellular sensing and responses to external materials that resemble the cell wall.

It is noteworthy to compare the cellular responses described here with those of a study looking at improving the production of secondary metabolites by Lindsey and coworkers [32]. The latter study took carrot and pepper suspension cultures, immobilised in polyurethane foam, and concluded that the immobilised cells have a metabolism that is closer to the respective whole plant – a useful property for industrial applications. Similar to our 3D culture, the polyurethane foam provides pores that are occupied by the cells, however, the polyurethane foam pores require a high seeding density for cells to be immobilised whereas the fibre scaffolds can immobilise single isolated cells. The foam-immobilised cells are also firmly attached since agitation does not dislodge them – an identical result to our 3D scaffolds. For carrot cells, the foam-immobilised cells can grow to a large size (up to 100 μm) and show a variety of shapes. Although these shapes do not achieve the complexity observed for our fibre scaffolds, the tight packing of the diverse carrot cells within the pores does resemble a simple intact tissue. Tight packing is observed in confined region of scaffold containing *Z. elegans* cells where cell growth and shape appeared largely homogenous. *Arabidopsis* cells are seen to grow past each other and no close packing or homogeneous growth of cells are observed for these cultures. The differences we observe between *Z. elegans* and *Arabidopsis* in scaffolds may be a function of (1) the higher seeding densities achieved with *Z. elegans*, which is 100x higher than *Arabidopsis*; and (2) the tissue the cells are derived from (*Z. elegans* cells are the mesophyll cells of leaves, whereas the *Arabidopsis* cells are from seed-derived calli).

The cytoskeleton, in particular the microtubules, are believed to play important roles in guiding the morphological changes of cells in response to the surrounding scaffold. Drugs that affect cytoskeleton function, such as oryzalin, latrunculin B and nocodazole, can be used in future investigations to better understand cell growth and morphological changes in scaffold-embedded culture media. Future work will also explore the possibility of targeting delivery of growth effectors such as hormones and cytoskeletal inhibitors by incorporating them directly into the scaffold. As a proof-of-concept, we have encapsulated the synthetic auxin, 2,4-D, within fibres. The next challenge is to better manage its release from the scaffold. This would potentially give rise to similar microgradients as found in the whole organism.

In summary, nano-structured scaffolds provide a powerful mechanism to encourage and direct cell behaviour ranging from cell adhesion to gene expression in animal tissue culture [3, 6, 33]. We envisage similar responses for plant cells leading to existing imaging, biochemical and genetic techniques being applied *in fibris*.

## Conclusions

We have developed a simple system that permits the study and facilitates imaging of fluorescently labelled cells interacting with a 3D environment. We have demonstrated that physical interactions with the local environment result in complex growth and morphogenesis.

## Methods

### Plant material

*Arabidopsis thaliana* lines expressing reporter constructs *35S::GFP-MBD* are used for visualising cortical microtubules, and *35S::GFP-FABD2* for visualising actin (gift from Tijs Ketelaar, Wageningen). Cytoplasmic mCherry is observed using a *35S::mCherry-TUA5* line that does not label microtubules (gift from Arun Sampathkumar, California Institute of Technology [34]). Lines containing *DR5::GFP-ER* and *PIN7-GFP* have been described previously [35, 36]. Seeds are surface sterilised for 15 minutes in sterilising solution consisting of 15 % Sodium Hypochlorite and 1 % Triton X-100 and washed 4 times in sterile water and finally resuspended in 4 volumes of sterile water. Seeds are vernalised at 4 °C for at least 48 hours before use in suspension culture.

The maintained cultured cells of *Arabidopsis thaliana* ecotype Colombia-0, used as a non-mucilage control, are a gift from Matthew Smoker (Sainsbury Laboratory, Norwich). Cells are maintained in MS liquid media containing Gamborg B5 vitamins and supplemented with sucrose (30 g l^−1^), 2-(n-morpholino)-ethanesulfonic acid (0.59 g l^−1^), 2,4-dichlorophenoxyacetic acid (1 mg l^−1^).

*Zinnia elegans* cells are propagated from mesophyll cells in non-inductive culture medium as described in Twumasi et al. [17]. Cells are dispensed in tubes containing pre-wetted scaffold at an initial density of 2 × 10^6^ cells ml^−1^.

### *Arabidopsis* cell culture preparation

Cell suspension cultures are prepared from *Arabidopsis* seed calli based on the protocol from Kevei et al. [37]. The culture medium consists of MS powder (Sigma M5524 4.32 g l^−1^), sucrose (30 g l^−1^), 2,4-dichlorophenoxyacetic acid (125 μg l^−1^), kinetin (15 μg l^−1^) and B5 vitamin stock (2 ml l^−1^ of stock consisting of 0.1 % w/v nicotinic acid, 0.1 % pyridoxine-HCl, 1 % thiamine-HCl and 10 % myo-inositol). Approximately 200 μl of surface sterilised and vernalised seeds are added to 40 ml culture media in a 500 ml flask followed by agitation at 130 rpm. Cultures are incubated at 22 °C until a suspension density of between 2 – 6 × 10^4^ cells ml^−1^ (7–21 days).

### Seeding cells to fibrous scaffolds

All procedures take place aseptically in a laminar air-flow cabinet. The scaffolds are pre-wetted in fresh culture medium and stirred with a sterile rod to remove trapped air. Scaffolds are placed in a Nunc® cryotube (Thermo Scientific cat no. 368632) towards the bottom placed at an oblique angle so that liquid can pass freely over and through the scaffolds during agitation. 1.5 ml of cell culture together with 0.5 ml of fresh MS medium is dispensed in a 2 ml CryoTube™. The scaffolds contain predominantly single cells than clumped cells and the quantity of cells within the scaffold varied between *Arabidopsis* lines and between experiments. The tubes are left to stand for 30 minutes, followed by placing them horizontally under agitation at 130 rpm. Cells are seeded in multiple scaffolds and at intervals, up until 11 days, one scaffold is removed for microscopy. To maintain the cells in scaffolds beyond 4 days, the culture medium needs to be replaced daily. Without this media change, lysed cells are observed by day 6, due to depletion of nutrients in the medium. The majority of the images were taken using scaffold-incubated cells taken between days 2 and 4 for a 2 ml culture without changing the MS medium.

### Live imaging of cells within scaffolds

For microscopic observations, the fibre scaffolds are either transferred to glass slides, maintained wet and observed with standard non-immersion objectives (without the addition of a cover slip); or for imaging at longer working distances, they are submersed in fresh growth medium, followed by imaging using a water-dipping objective. High-resolution 3D imaging and time lapse 4D imaging typically involve timescales between 5 and 45 minutes, during which multiple frames are averaged using the frame averaging feature in the imaging software to improve the signal to background noise ratio.

Live imaging is carried out using a Zeiss LSM700 confocal microscope, a Zeiss LSM780 confocal microscope or a Zeiss Axioimager M2 optical microscope fitted with DIC optics. For immediate imaging with the 20x NA 0.8 plan-apochromat non-immersion objective, scaffolds are removed from tubes and placed flat on a glass slide. To achieve longer working distances at higher resolution, scaffolds are placed in a small container or petri dish and weighed down at the edges using steel razor blades and covered with growth media. A 20x NA 1.0 water-dipping objective is then carefully lowered into the liquid. Multi-dimensional acquisition is carried out using Zen software where a z-spacing of between 0.4 – 1 μm is used for acquiring stacks. Both GFP and fibre fluorescence are acquired simultaneously using 488 nm excitation and emission collected at between 495 and 555 nm (GFP) and between 560 and 735 nm (for fibre autofluorescence). mCherry, together with fibre autofluorescence are visualised using 555 nm excitation and emission collected between 560 and 735 nm.

### Scanning electron microscopy

Scanning electron microscopes (models: FEI Nova NanoSEM™, Zeiss EVO HD15) are used to analyse the dried samples. In addition, a focused ion beam (FIB) electron microscope (FEI Helios) is used to image and cut the samples. For the FEI SEMs, each SEM sample is coated with gold using a sputtering machine (Emitech K550, Emitech Ltd, UK) for 120 s prior to observation. The coating is approximately 15 nm in thickness. For the Zeiss EVO HD, samples are placed directly on the stage without processing. Gun emission is set to 10–20 kV. All images are acquired using the backscatter detector. Variable Pressure (VP) imaging is carried out at 40 Pa. Due to the apparently delicate nature of the cells from the long-term line (non-mucilage control), highly deflated cells are observed under VP mode.

Films are sometimes observed in the SEM images, (e.g. the top corner of Fig. 3b). These films are attributed to the MS culture medium, as the same films are also seen in scaffolds agitated in MS medium without cells.

### Image processing

The micrographs are analysed using ImageJ, a public domain Java image-processing program. The tonal range and colour balance of the images are optimised to sharpen the contrast of the fibres and cells against the background, using the levels histogram in photoshop to adjust intensity levels of shadows, midtones, and highlights.

### 3D fibre scaffolds

Shear-spun fibrous scaffolds as dry thick discs (>50 μm thick) of interlocking homogeneously entangled microfibres with nanofibres (Xanofi Ltd., North Carolina, USA) are used. The scaffolds consist of PLA nanofibres and PET microfibres. Little is known about scaffold biocompatibility requirements for plant cells and how the cells interact with a scaffold. PLA–PET scaffolds are used as they are commercialised 3D scaffolds made by shear spinning, which is under investigation for tissue engineering in our laboratory.

The ability to achieve uniform nanofibre dispersion and control of porosity in a bulk scaffold is a unique feature of the shear spinning method. Other nanofibre-forming techniques, including electrospinning, can only achieve dense and thin sheets with nanometre-pores and micrometre-thickness, leaving cells unable to move through the pores; shear-spun scaffolds can provide various nanofibre percentages while maintaining the same porosity profile.

Three different scaffolds with varying percentages by weight of nanofibre to microfibre ratios but similar porosity profiles are tested to observe the influence of nanofibres percentage on cell seeding efficiency and growth profile. The scaffold types are 100 % PET microfibres; 10 % PLA nanofibres, 90 % PET microfibres; and 30 % PLA nanofibres, 70 % PET microfibres. The scaffold material is cut to 10 mm × 10 mm pieces prior to sterilisation treatment and cell seeding.

The porosity of the scaffold of each percentage ratio of nano- and microfibres are characterised. Specifically, the mass of a dry scaffold, *m*, is measured using a digital balance. The width, *w*, and length, *L*, of the scaffold are measured using a digital caliper. The thickness of the scaffold (*h*) is imaged by SEM. Three measurements are taken per sample and the average values recorded. The porosity is calculated using the following equation: Porosity (%) = [(1 − ad)/bd] × 100. Where, ad is the polymer(s) apparent density (gcm^−3^), calculated as ad = *m*/(*h* × *w* × *L)*; bd is the bulk density of pure amorphous polymer(s) prior to fibre formation. The amorphous densities of the polymers are provided by the supplier Xanofi Ltd, respectively 1.24 g cm^−3^ and 1.38 g cm^−3^ for PLA and PET polymers. The average cell count per mm^2^ is calculated based on 3 samples per type of scaffold, over an area of 2 mm^2^ per sample.

### Encapsulation of auxin

Synthetic auxin, 2,4-dichlorophenoxyacetic acid (2,4-D, C_8_H_6_Cl_2_O_3_) is dissolved (0.5 % w/w) in 15 % w/w PLA in chloroform/methanol 3:1 v/v solution. The resultant mixture is shear-spun into microfibrous scaffolds and X-ray sterilised for cell culture. Shear spinning process has been previously described [10]. Control studies are carried out in parallel, using (1) cell suspension with no scaffold, (2) cells in scaffolds made by the same method but without auxin.

### Optimising sterilisation methods for the scaffolds

#### Ethanol treatment

The scaffold is immersed in 100 % ethanol for 2 hours and stored in a sealed Petri dish. The ethanol-treated scaffold is immersed in cell culture medium for 10 minutes in laminar air-flow cabinet prior to cell seeding. Some residue ethanol in the scaffold is possible.

#### Ultraviolet irradiation

The scaffolds are placed in a Petri dish of 12 mm diameter. The polystyrene Petri dish is sealed with Parafilm® and placed on aluminium foil. Irradiation is carried out with a ultraviolet lamp (8 W, 3UV™-38, UVP, Cambridge, UK) at a wavelength of 254 nm and a distance of 50 mm. Samples are irradiated for a total time of two hours, and are turned over halfway through the treatment to irradiate the top and bottom surfaces.

#### X-ray irradiation

Dry scaffolds are placed in CryoTube™ vials (Thermo Scientific, UK), and placed in the centre of the X-Ray chamber (0.5 mm Cu filter, 220 kV, 14 mA, Pantak PMC1000). Each scaffold is irradiated at a dose of 417 cGy/min for 18 minutes.

## Additional files

Additional file 1: Figures S1-S9.
**Figure S1.** Projections of confocal z-stacks of Arabidopsis thaliana cells containing the microtubule reporter construct *35S::GFP-MBD*: (a-b) immediately before seeding into scaffolds; (b-e) at day 2, 6 and 11 of scaffold incubation. Scale bars: 100 µm. **Figure S2.** Scaffold morphology observed under SEM (a) before treatment, (b) after UV treatment, (c) after 18 min X-Ray treatment, (d) after immersion in ethanol for 2 hr. Extensive nanofibre fusion is observed in (d). Scale bars: 400 µm. **Figure S3.** SEM image of a seeded scaffold incubated in growth medium under constant agitation. Arrows indicate small, round and immobilised cells of 47 ± 8 µm. Scale bar: 500 µm. **Figure S4.** Silica beads (diameter range 40–200 µm, 2.5 x 10^4^ beads per ml^-1^ in MS growth medium) trapped in the scaffold (a) before and (b) after constant agitation at 130 RPM for 3 days. Scale bars: 100 µm. **Figure S5.** DIC microscopy of a cell culture free from seed-mucilage contamination. Images show a cell wrapping around a microfibre and interacting with neighbouring fibres. Depth of view: (a) 0 µm, (b) 7.2 µm. Arrows indicate cell-fibre interaction. Red lines highlight relevant microfibres. Scale bars: 100 µm. **Figure S6.** Spiral growth of a cell around a microfibre. Red lines highlight locations of microfibers. (a) Actin reporter (b) transmission. Scale bars: 100 µm. **Figure S7.** Confocal z-projections showing GFP-labelled microtubule patterns in *A. thaliana* cells expressing the reporter construct *35S::GFP-MBD*. White arrows indicate microtubules aligned parallel with the principal growth direction. Main panel scale bar: 100 µm. **Figure S8.** Confocal (a-b, showing autofluorescent cells and microfibres) and high vacuum SEM (c-d, greyscale) images of mesophyll cells of *Zinnia elegans* cultured in scaffolds at day 3 after seeding. Arrows indicate cell-fibre interactions. Scale bars: 100 µm. **Figure S9.** Arabidopsis cells expressing *DR5::GFP-ER* in unmodified scaffolds (a GFP, b transmission, cell outlines are indicated by arrowheads) and in scaffolds encapsulated with the synthetic auxin 2,4-D (c). Scale bars: 100 µm (d) Cell-scaffold PIN7-GFP is observed in discrete punctae (arrows) and the localisation does not differ from cells in liquid culture (data not shown). Scale bar: 10 µm. Additional file 2:
**An Apple Quicktime movie named Movie S1.mov 820 showing a 3D reconstruction of confocal z-stack of microtubule 821 organisation in a rounded cell existing and interacting between two 822 individual fibres.** (MOV 5686 kb) Additional file 3:
**A Windows Media movie Movie S2.avi showing a 824 4D dataset of microtubule dynamics.** White arrow shows the plus end 825 of an individual microtubule undergoing rounds of growth (polymerisation) 826 and catastrophe (depolymerisation). (AVI 699 kb)
