# Supplementary material for: A 3-dimensional fibre scaffold as an investigative tool for studying the morphogenesis of isolated plant pells
Source: BMC Plant Biol. 2015 Aug 26;15:211. doi: 10.1186/s12870-015-0581-7 (PMC4550058; doi:10.1186/s12870-015-0581-7)
Supplement: Additional file 1: Figures S1-S9. — Figure S1. Projections of confocal z-stacks of Arabidopsis thaliana cells containing the microtubule reporter construct 35S::GFP-MBD: (a-b) immediately before seeding into scaffolds; (b-e) at day 2, 6 and 11 of scaffold incubation. Scale bars: 100 µm. Figure S2. Scaffold morphology observed under SEM (a) before treatment, (b) after UV treatment, (c) after 18 min X-Ray treatment, (d) after immersion in ethanol for 2 hr. Extensive nanofibre fusion is observed in (d). Scale bars: 400 µm. Figure S3. SEM image of a seeded scaffold incubated in growth medium under constant agitation. Arrows indicate small, round and immobilised cells of 47 ± 8 µm. Scale bar: 500 µm. Figure S4. Silica beads (diameter range 40–200 µm, 2.5 x 10^4^ beads per ml-1 in MS growth medium) trapped in the scaffold (a) before and (b) after constant agitation at 130 RPM for 3 days. Scale bars: 100 µm. Figure S5. DIC microscopy of a cell culture free from seed-mucilage contamination. Images show a cell wrapping around a microfibre and interacting with neighbouring fibres. Depth of view: (a) 0 µm, (b) 7.2 µm. Arrows indicate cell-fibre interaction. Red lines highlight relevant microfibres. Scale bars: 100 µm. Figure S6. Spiral growth of a cell around a microfibre. Red lines highlight locations of microfibers. (a) Actin reporter (b) transmission. Scale bars: 100 µm. Figure S7. Confocal z-projections showing GFP-labelled microtubule patterns in A. thaliana cells expressing the reporter construct 35S::GFP-MBD. White arrows indicate microtubules aligned parallel with the principal growth direction. Main panel scale bar: 100 µm. Figure S8. Confocal (a-b, showing autofluorescent cells and microfibres) and high vacuum SEM (c-d, greyscale) images of mesophyll cells of Zinnia elegans cultured in scaffolds at day 3 after seeding. Arrows indicate cell-fibre interactions. Scale bars: 100 µm. Figure S9. Arabidopsis cells expressing DR5::GFP-ER in unmodified scaffolds (a GFP, b transmission, cell outlines are indicated [file 12870_2015_581_MOESM1_ESM.pdf]

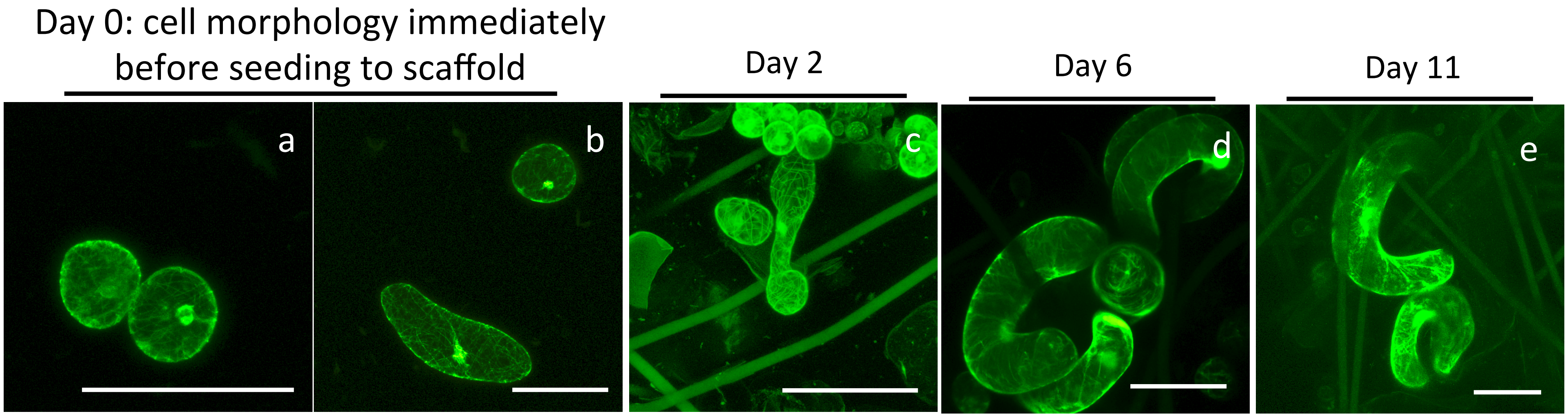
Day 0: cell morphology immediately before seeding to scaffold
Day 2
Day 6
Day 11
a
b
c
d
e

Figure S1

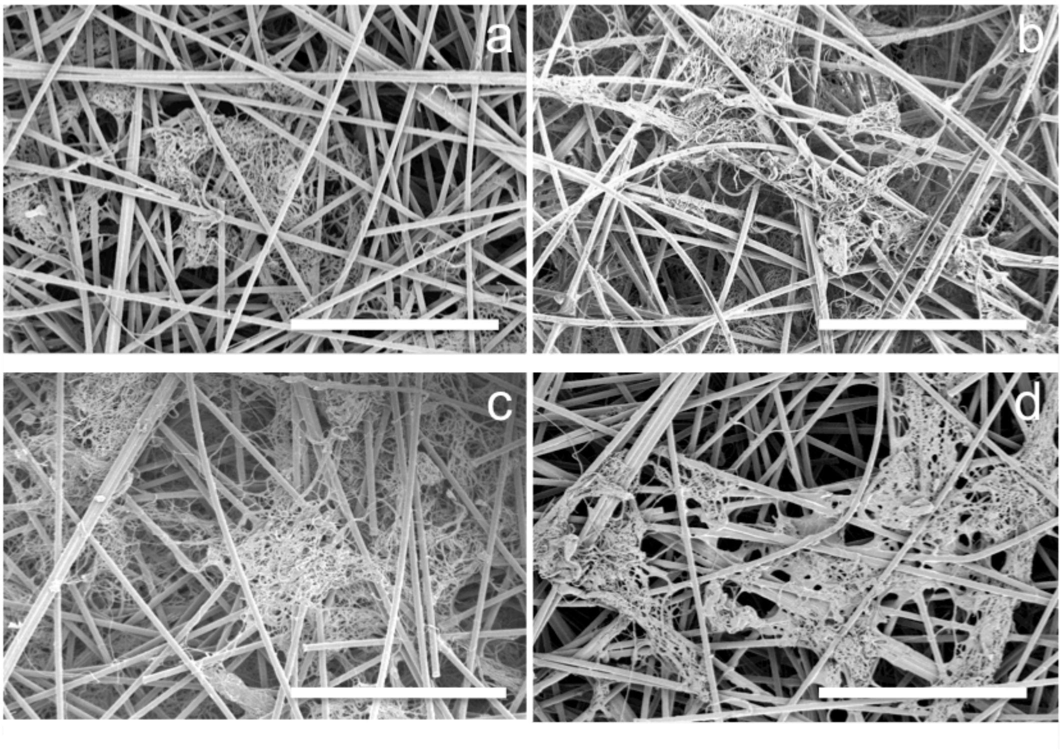

Figure S2

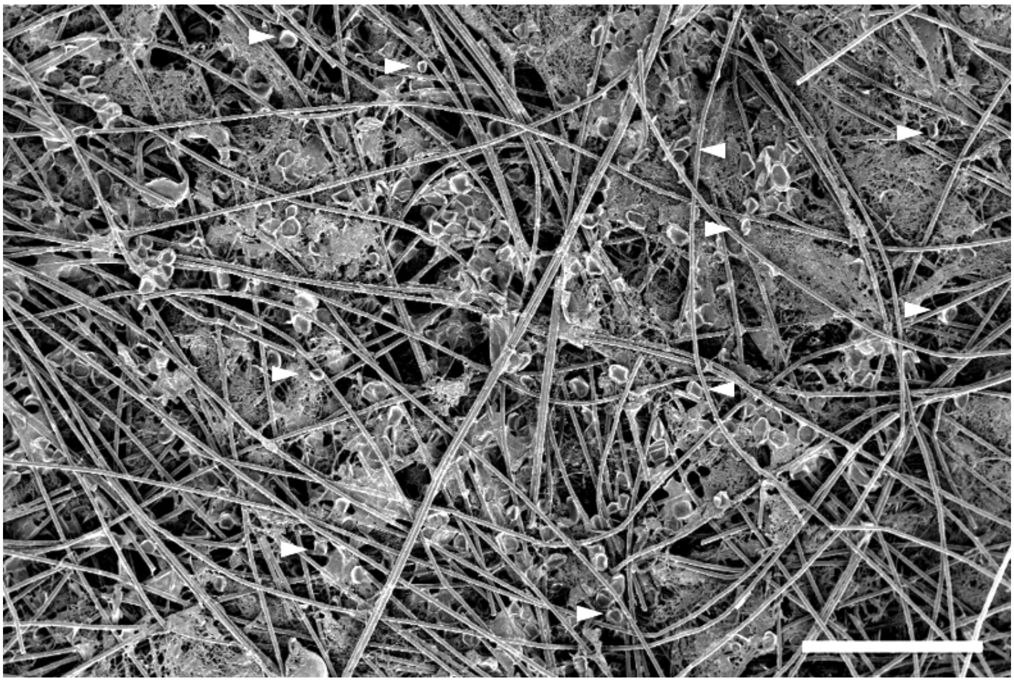

Figure S3

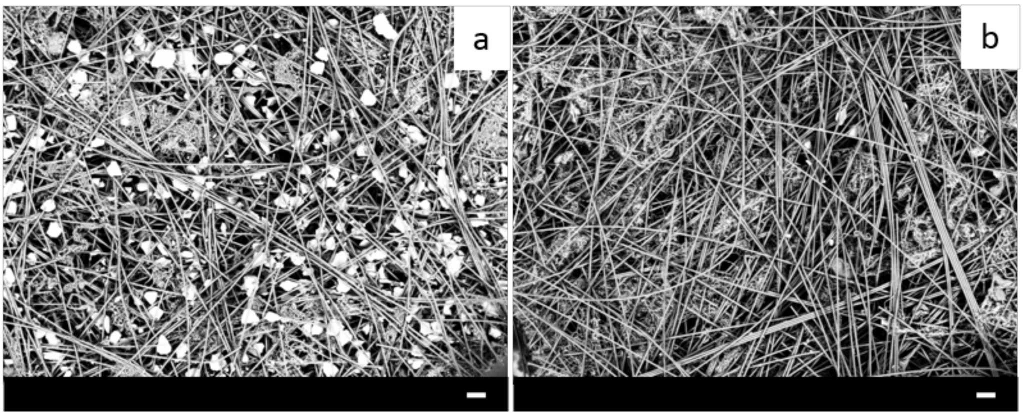

Figure S4

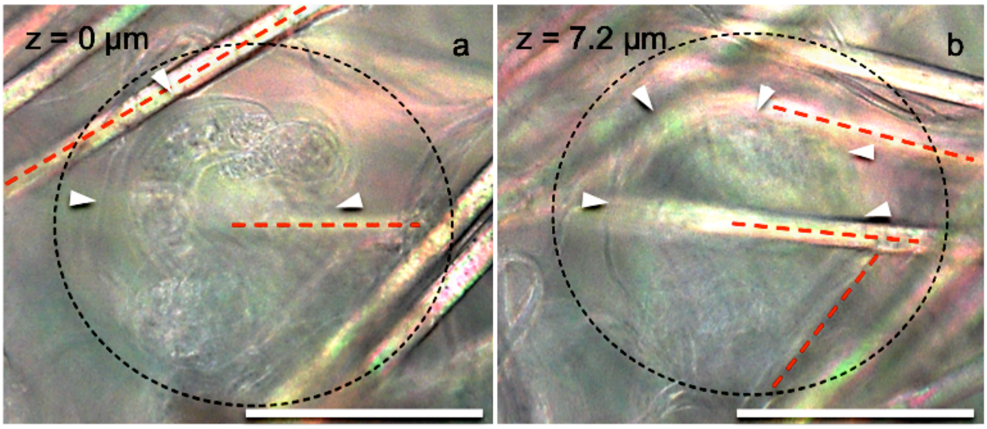

Figure S5

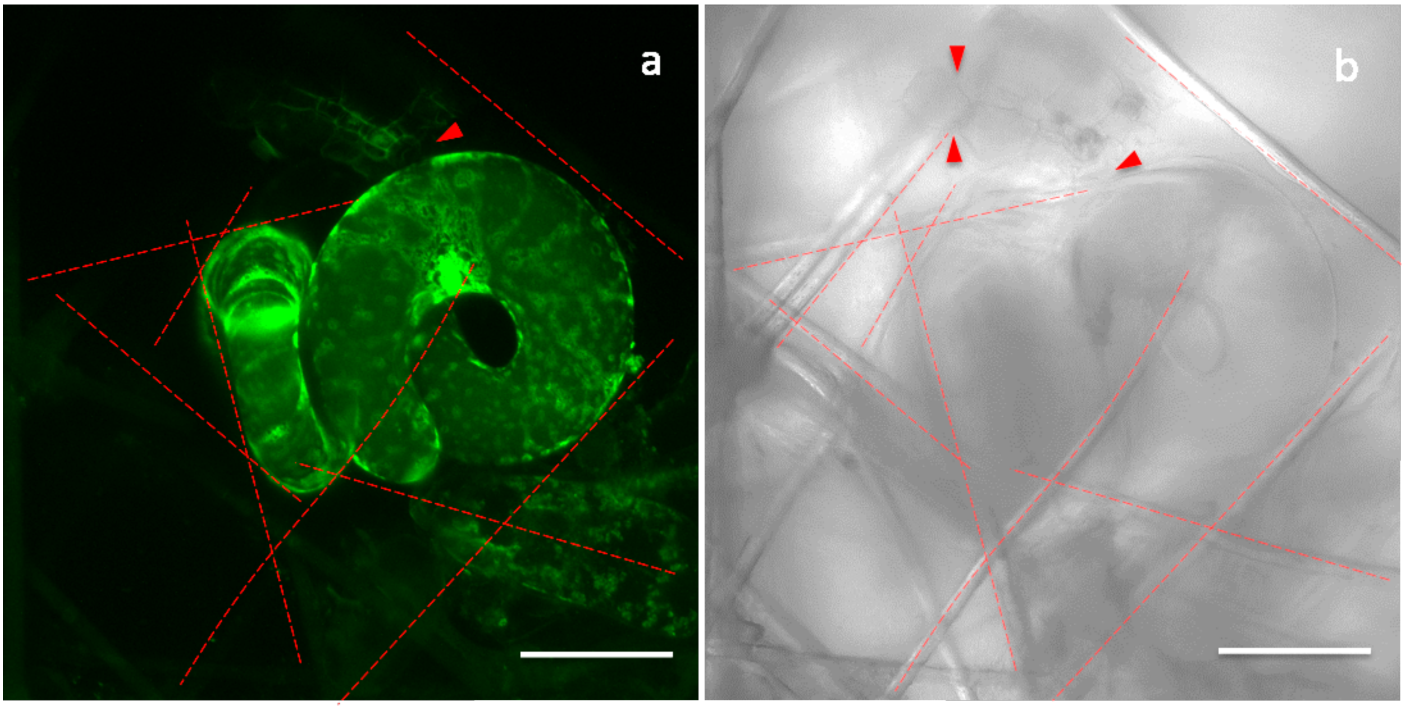

Figure S6

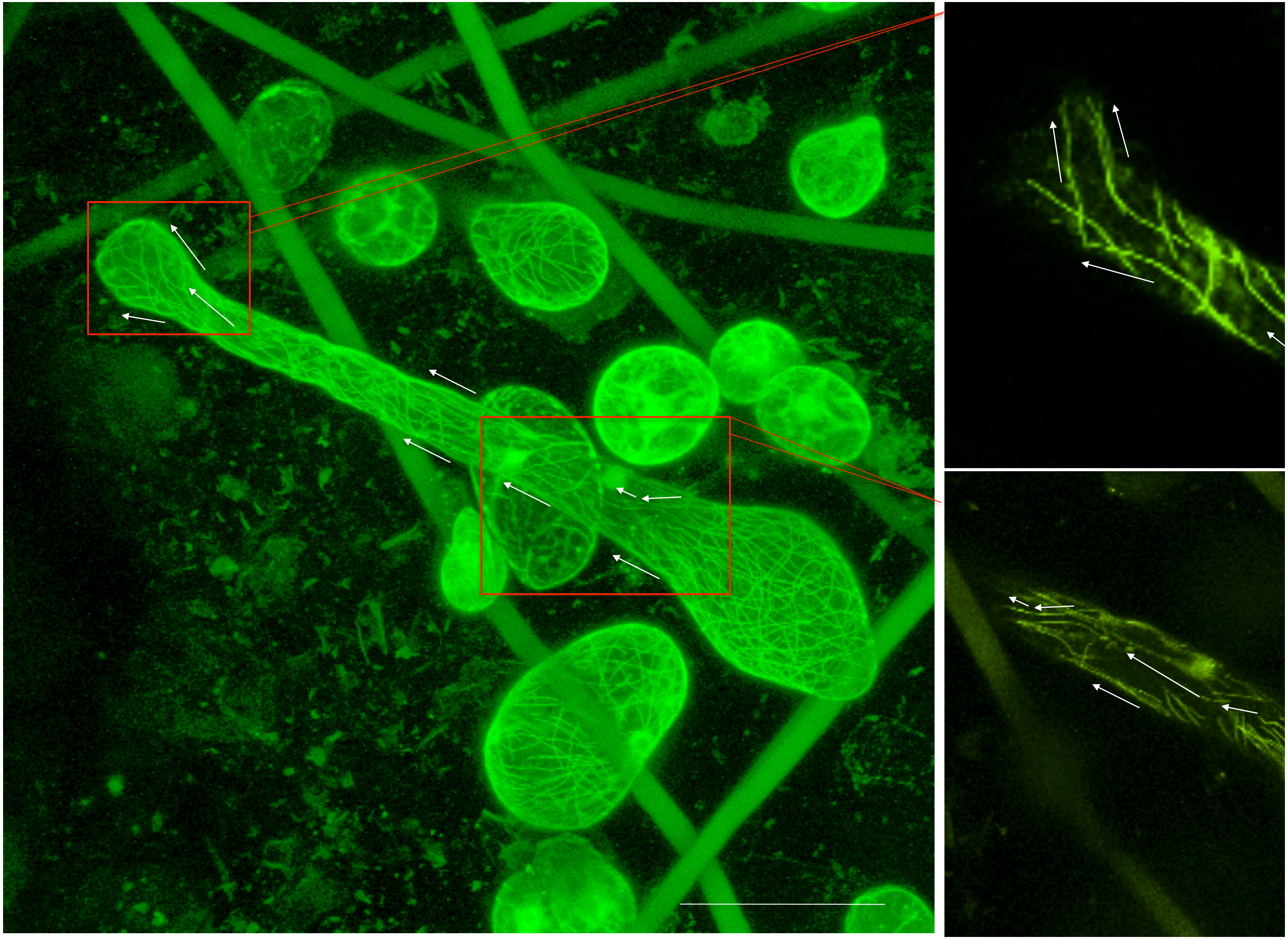

Figure S7

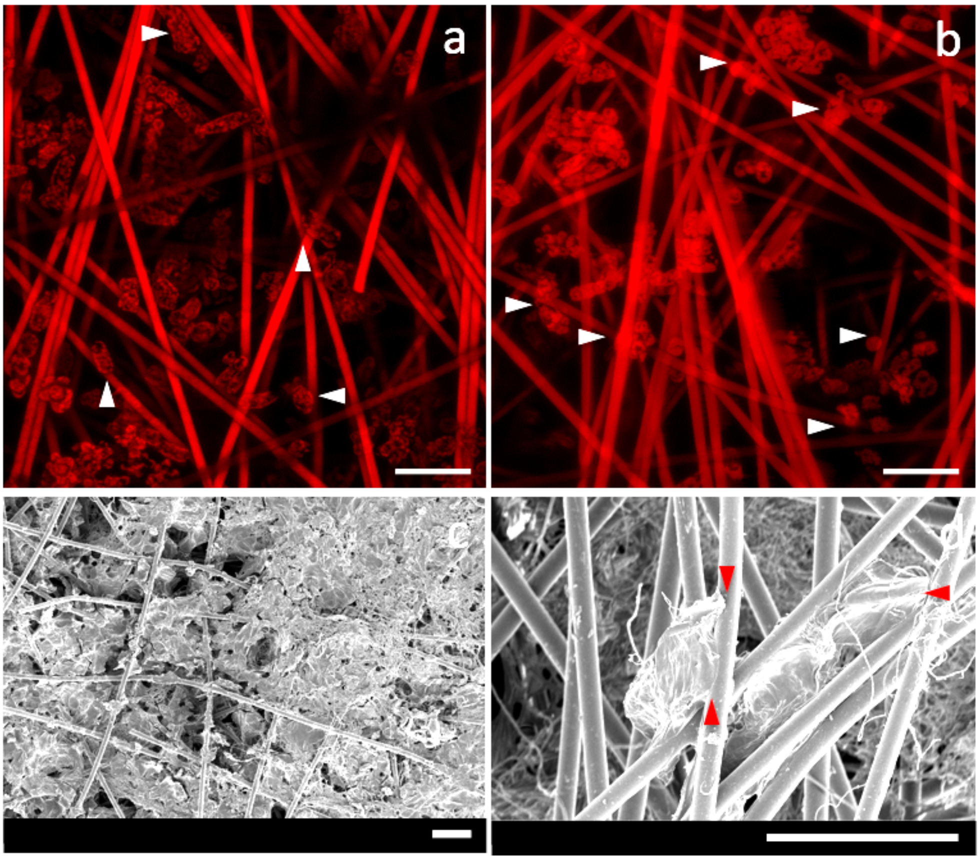

Figure S8

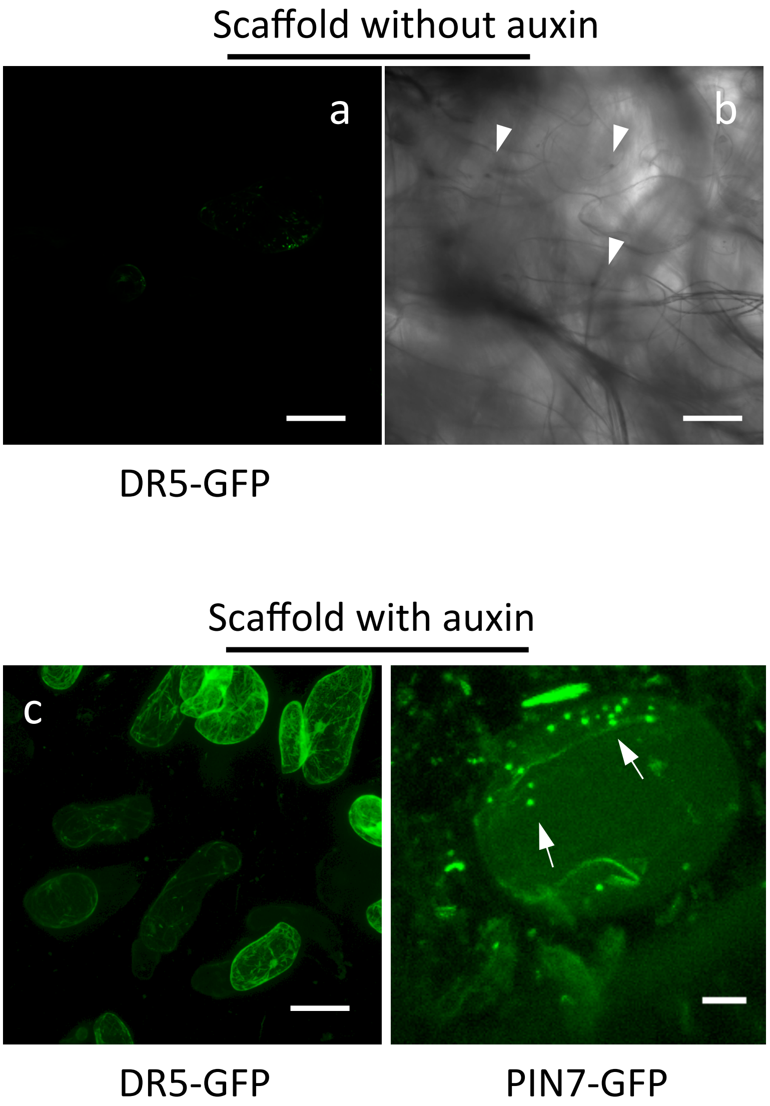

Scaffold without auxin
a
b
DR5-GFP
Scaffold with auxin
c
DR5-GFP
PIN7-GFP

Figure S9
